# A Rise in Measles Reporting Two Years After the COVID-19 Pandemic: A Descriptive Analysis of Measles in Iraq in 2023 and Early 2024

**DOI:** 10.3390/ijerph23060760

**Published:** 2026-06-05

**Authors:** Hanan Abdulghafoor Khaleel, Riyadh Abdulameer Alhilfi, Sabrina Viele Brown

**Affiliations:** 1 Communicable Diseases Control Centre, Directorate of Public Health, Ministry of Health, Baghdad 10069, Iraq; hanan.abdulghafoor@gmail.com; 2 Directorate of Public Health, Ministry of Health, Baghdad 10047, Iraq; riyadalhilfi@gmail.com; 3 Department of Epidemiology, Kentucky Injury Prevention and Research Center, College of Public Health, University of Kentucky, Lexington, KY 40536, USA

**Keywords:** measles, Iraq, vaccine-preventable, clusters

## Abstract

**Highlights:**

**Public health relevance—How does this work relate to a public health issue?**

- Although reported measles cases in Iraq declined between 2020 and 2022, a substantial global resurgence of measles began in late 2022.
- These trends emphasize the need for continued measles control efforts and epidemiologic analyses to inform prevention strategies. A worldwide surge in cases started in late 2022.

**Public health significance—Why is this work of significance to public health?**

- The primary aim of this study was to identify spatial clusters of high and low measles incidence, a critical step in understanding the geographic heterogeneity of measles transmission.
- Specifically, the aim was to identify high-incidence clusters to inform targeted prevention and control efforts, as well as low-incidence clusters to explore potential protective factors.

**Public health implications—What are the key implications or messages for practitioners, policy makers and/or researchers in public health?**

- Identification of high-incidence clusters can support targeted vaccination campaigns, enhanced surveillance, and focused risk communication in priority areas.
- Low-incidence clusters may provide insights into effective practices, resilience, or protective contextual factors that could be adapted to other settings.

**Abstract:**

**Background:** The elimination of measles is a public health priority for the World Health Organization. During the COVID-19 pandemic from 2020 to 2022, the number of cases in Iraq decreased. However, a surge in cases started in late 2022. The aims of this study are to understand and describe the epidemiology of the surge of measles compared to reported cases in 2018 and 2019. Secondarily, they are to identify high clusters to find possible causes and implement prevention efforts accordingly, and low clusters of measles to identify possible protective factors. **Methods:** Frequencies were used to describe the univariate characteristics of cases reported each year. The chi-square test of independence was used to test differences by age; *p*-values less than 0.05 were considered statistically significant. Gi cluster analysis was used to determine where there were high and low clusters of cases in each district. **Results:** The number of clinically confirmed cases of measles rose dramatically in 2023 (14,301) and early 2024 (33,048) compared to 2018 (1044) and 2019 (4586). Most patients were less than one year to 14 years of age. The percentage of patients aged 5–14 years was higher in 2023 (32.8%) and 2024 (30.0%) than in 2018 (15.9%) and 2019 (22.1%). Males were consistently more prevalent than females throughout all study years. Almost 5% (1545) of patients were vaccinated; the remainder were unvaccinated or had unknown vaccination status. Only 1% reported a history of contact with infected patients. The case fatality ratio was 0.06% in 2023 and 0.2% in early 2024. Despite the recent surge in cases, 27 of the 153 districts (17.4%) had low clustering. **Conclusions:** The recent surge in measles cases in Iraq was found to be in those below 15 who are commonly associated with the disease. Clusters of high reporting were mainly in the middle of Iraq while clusters of low reporting were mainly in the north. We recommend continuing to study clusters of measles and vaccine coverage to direct prevention efforts.

## 1. Introduction

The elimination of measles is a public health priority of the World Health Organization (WHO) [1]. The elimination appeared attainable following the development of a more effective measles vaccine and the combined measles, mumps, and rubella (MMR) vaccine [2]. During the COVID-19 pandemic there were low numbers of cases (estimated to be less than 1/100,000 population). There were four cases in 2020; in 2021 there were no cases and in 2022 there were 86 cases. The surge of cases started late in 2022 and continued through 2023. To date, there have been no epidemiological studies describing trends, patterns, or clusters of the recent surge in measles cases in Iraq.

Measles is a highly communicable airborne disease caused by an RNA Morbillivirus of the Paramyxoviridae family [3]. Measles is also preventable with vaccination. The WHO initially targeted the elimination of measles by 2020. Despite the presence of multiple effective vaccines against measles, eradication still poses a major worldwide public health challenge due to vaccine reluctance, misinformation about the possible vaccine side effects, lack of awareness about the consequences of measles infection, gaps in routine immunization programs, delays in responding to outbreaks, clusters of unvaccinated individuals, insufficient resources to achieve 95% two-dose vaccine coverage at the national and subnational level, and variation in the immune response to the circulating clade [2,3,4]. Therefore, the WHO has extended the elimination deadline to 2030 [1].

According to the WHO, the number of measles cases has increased by 66.5% in 2022 from what was recorded in 2021 globally [5]. In fact, the increase in cases was across all regions of the WHO except regions of the Americas where there was a decrease of 93.1% [5]. The Eastern Mediterranean Region ranked third in the rise (116.2%) after the European Region (760.6%) and the Southeast Asia Region (663.0%) [5]. The top three countries in the Eastern Mediterranean Region that showed a dramatic rise in the number of measles cases in 2022 and 2023 were Iraq (1026.5%), Qatar (126.0%), and Egypt (18.07%) [6].

Additional key drivers of the measles resurgence include the virus’s high transmissibility, insufficient vaccine coverage to maintain herd immunity, and waning adherence to non-pharmaceutical public health measures [3].

According to the annual reports of the Iraqi Ministry of Health, the number of measles cases declined in 2020–2022 [6,7]. However, there was a surge of cases in 2023 and early 2024 (1 January–19 May) to levels higher than what was reported before the pandemic. The resurgence of measles in Iraq is not an isolated phenomenon. Globally, measles cases surged dramatically in the first five months of 2024, with an estimated 11 million infections worldwide—nearly 800,000 more than pre-pandemic levels in 2019—and approximately 95,000 deaths, mostly among children under five years of age, according to a recent WHO report [8,9]. In 2024, 59 countries reported large or disruptive measles outbreaks, nearly triple the number reported in 2021 and the highest since the onset of the COVID-19 pandemic [10]. All WHO regions except the Americas had at least one country experiencing a large outbreak in 2024, though the situation worsened in the Americas in 2025 [10].

In the WHO Eastern Mediterranean Region, which includes Iraq, measles cases increased by 86% in 2024 compared with 2019 [10,11]. Provisional data from the CDC covering October 2023 to March 2024 ranked Iraq as the country with the third-highest number of measles cases globally during that period, with 25,429 reported cases, behind only Azerbaijan (28,787) and Kazakhstan (28,660) [12]. This regional surge mirrors the global trend and underscores the widespread nature of the measles resurgence. Prior to this study there was no description of the characteristics and geographic distribution to help in characterizing the cases epidemiologically to help in planning and directing public health services.

Collectively, these global and regional data demonstrate that the measles resurgence following the COVID-19 pandemic has been a widespread phenomenon, affecting countries across all income levels and geographic regions. Iraq’s experience is thus part of a broader pattern of post-pandemic measles resurgence, driven by common factors such as disruptions to routine immunization, waning population immunity, and challenges in achieving the 95% vaccination coverage threshold required for herd immunity. Understanding the epidemiological characteristics of the surge in Iraq—including its demographic patterns and geographic distribution—is therefore essential not only for guiding local public health responses but also for contributing to the global understanding of the post-pandemic measles dynamics.

Our primary aims in this study are to describe the epidemiology of measles in the recent surge in 2023 and early 2024, compare the demographic trends of the recent surge with the demographics of the cases reported in 2018 and 2019, and identify clusters of high and low reporting in 2024.

## 2. Methods

Study design: A retrospective review and analysis of surveillance data in Iraq to describe the differences in the demographics of cases of suspected measles reported in 2018, 2019, 2023, and the first five months of 2024 (1 January to 19 May).

Study population, data source, and surveillance system: All residents of Iraq who developed the signs and symptoms of measles and attended a governmental primary healthcare center or hospital to seek healthcare and diagnosis were reported immediately to the surveillance section as “suspected Measles and/or Rubella”as shown in Table 1. Serum and urinary samples from each patient were collected and sent to the Central Public Health Laboratory in Baghdad to test for IgM Ab for measles and IgM Ab and IgG Ab for rubella using ELISA. Further molecular tests were done on a number of the urinary samples of the patients who tested positive to identify the genotype. Duplicates were excluded.

Cases were investigated; officials in each district were notified, including the surveillance officer at the Department of Health and the surveillance section at the Communicable Diseases Control Center at the Public Health Directorate at the Ministry of Health. The surveillance section collects case-based information about each suspected case of measles from 20 Departments of Health (DsoH) all over Iraq. Each DoH collects data from district repositories containing information from surveillance sites (primary healthcare centers and hospitals). The number of districts is subject to change based on administrative changes. We used the 2019 district counts (153 districts) to calculate and map the incidence per 100,000 population.

Since 2019, measles has been a case-based immediately notifiable disease in Iraq; a notification form is collected electronically via EpiInfo 7.2 software. Surveillance officers at the DsoH receive the laboratory results from the Central Public Health Laboratory (CPHL), update the final diagnosis, and resend to the surveillance section.

Variables: The variables of this study included year (2018, 2019, 2023, early 2024 (up to May 19)), diagnostics (clinical, laboratory-confirmed, Epi-linked), sex (male, female), age groups (younger than 1 year, 1–4 years, 5–14 years, 15+ years), vaccination status (vaccinated, unvaccinated or unknown vaccination status), history of contact (yes, no/unknown), outcome (deceased, recovered). Vaccination status was categorized as vaccinated (documentation of at least one dose of a measles-containing vaccine administered ≥14 days before rash onset), unvaccinated (no documented doses), or unknown (missing or unclear vaccination records). The national immunization schedule for measles is to give MCV at 9 months, 1st dose of MMR at 12 months, and 2nd dose MMR at 18 months.

Death occurring within 30 days of rash onset or death attributed to measles or its complications, as determined by the treating physician or surveillance officer, was updated in the system.

Statistical analysis: We analyzed surveillance data of suspected measles and/or rubella cases reported in 2018, 2019, 2023, and early 2024. Because the number of confirmed and clinical cases in 2020, 2021, and 2022 were so low, we did not include them in the mapping. Frequency and percentages were used to describe the univariate characteristics of the cases reported each year. The chi-square test of independence was used to test the differences between the demographics in 2023 from 2018 and 2019 and *p*-values less than 0.05 were considered statistically significant. Microsoft Excel was used to derive pivot tables, frequencies, and percentages, as well as calculate the chi-square test of independence to test for the relation between age groups and years. Cases with unknown vaccination status or not vaccinated were included in the chi-square analysis as a category.

We used Getis-Ord Gi* statistic (Gi*) to determine clusters of high- and low-reporting districts. A queen contiguity spatial weights matrix was used to define neighbors, where districts sharing a common border or vertex were considered neighbors. The Gi* resultant z-scores and *p*-values determine where features with either high or low values cluster spatially through looking at each feature within the context of neighboring features. To be a statistically significant hot spot, a feature will have a high value and be surrounded by other features with high values as well. The local sum for a feature and its neighbors is compared proportionally to the sum of all features; when the local sum is very different from the expected local sum, and when that difference is too large to be the result of random chance, a statistically significant z-score results. For statistically significant positive z-scores, the larger the z-score, the more intense the clustering of high values (hot spot). For statistically significant negative z-scores, the smaller the z-score, the more intense the clustering of low values (cold spot). We used GeoDa software v. 1.22.0.2 to calculate Gi* statistics and generate maps of measles incident cases per 100,000 population, by districts.

## 3. Results

**Figure 1:**
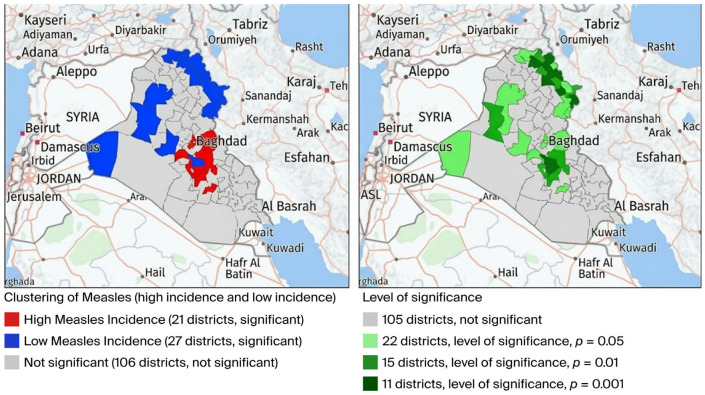
Clustering of measles and statistical level of significance.

The number of clinically confirmed cases of measles rose dramatically in 2023 (14,301) and early 2024 (33,048) compared to 2018 (1044) and 2019 (4586). When adjusted for population size, measles incidence increased from 2.6 per 100,000 population in 2018 to 11.1 per 100,000 in 2019 and rose sharply to 31.7 per 100,000 in 2023 and 71.8 per 100,000 in early 2024. These population-adjusted rates confirm that the observed rise in measles cases reflects a true increase in disease burden rather than population growth alone (please refer to Table 2 and Table 3 and Figure 1).

Males were consistently slightly more prevalent than females throughout the study years. Just under 5% of the patients were vaccinated; the remainder were either unvaccinated or with unknown vaccination status. Only 1% reported a history of contact with the infected patients. The case fatality ratio was 0.06% in 2023 and 0.2% in early 2024.

Despite minor differences across the years, most patients were less than one year, 1–4 years, and 5–15 years. The percentage of patients aged 5–15 years was higher in 2023 (32.8%) and 2024 (30.0%) than in 2018 (15.9%) and 2019 (22.1%). A chi-square test of independence showed statistical significance between age groups and years (X2 = 1111.2463, *p* =< 0.00001). The results from the chi-square test between years and age groups indicate that there is a statistically significant association between the two. We did not compare the difference in proportion in one age group across the years.

## 4. Discussion

Findings from our study show that the numbers of clinical and confirmed cases of measles have risen dramatically in 2023 (14,301) and early 2024 (33,048) compared to 2018 (1044) and 2019 (4586). The decline in the numbers of cases in 2020 and 2021 may have been due to the non-pharmaceutical public health interventions (NPPHI), such as social distancing, online education, curfews, and face mask mandates implemented during the pandemic which reduced the occurrence of most communicable diseases, including measles [13,14]. Conversely, the communities’ attitude toward vaccines, as a backlash against the COVID-19 vaccinations, may have led to an increase in susceptible individuals and lessening restrictions may have facilitated the transmission of measles, especially given the high reproduction rate of measles [14]. Other worldwide challenges to the elimination of measles are vaccine reluctance because of misinformation about the possible vaccine side effects, lack of awareness about the consequences of measles infection, gaps in routine immunization programs, delays in responding to outbreaks, clusters of unvaccinated individuals, insufficient resources to achieve two-dose vaccine coverage at the national and subnational level, and variation in the immune response to the circulating clade [2,3,4].

The surge in cases is not limited to Iraq but is worldwide, including developed countries and countries close to elimination [13,15,16]. Globally, measles resurged dramatically in 2024, with an estimated 11 million cases and approximately 95,000 deaths, marking a return to pre-pandemic levels of transmission [9]. The WHO reported that 59 countries experienced large or disruptive outbreaks in 2024, nearly triple the number in 2021 and the highest since the onset of the COVID-19 pandemic [10]. In Europe and Central Asia, more than 127,350 cases were reported—double the 2023 total and the highest annual figure in over 25 years—with Romania (30,692 cases), Kazakhstan (over 28,000), and the United Kingdom (over 2900 cases) among the most affected [13,15]. In the Eastern Mediterranean Region, cases increased by 86% compared with 2019, with Iraq (25,429 cases between October 2023 and March 2024), Yemen, and Pakistan reporting substantial outbreaks [11,12]. Across Asia, India, Indonesia, and Vietnam, all experienced major resurgences, with Vietnam alone recording over 45,000 suspected cases by late 2024 [17,18]. In the Americas, the region initially fared better but saw a sharp rise in 2025, with Canada losing its measles-elimination status for the first time in 25 years following a large outbreak that began in late 2024 [16,19]. Collectively, these data demonstrate that the post-pandemic measles resurgence has been a global phenomenon, driven by persistent immunity gaps, disruptions to routine immunization, vaccine hesitancy, and fragile health systems [20,21].

Measles was nearly eliminated in the United States but has seen resurgences. Texas had its most severe outbreak in nearly three decades in early 2025 [2]. In Canada, an unprecedented rise in incidence was observed, particularly in Ontario and Alberta—more than thirtyfold higher than the national average. In Mexico, the outbreak was highly geographically concentrated. Italy experienced a marked escalation in measles cases in 2024 compared with prior years, predominantly among unvaccinated individuals. Sustained transmission was documented in major metropolitan regions, including Lazio and Lombardy [2,4].

Despite minor differences in the age group distribution throughout our study, most of the patients were younger than 1 year, 1–4 years, and 5–14 years. The percentage of patients aged 5–14 years was higher in 2023 (32.8%) and 2024 (30.0%) than in 2018 (15.9%) and 2019 (22.1%). This finding may be due to gaps in the immunization programs in 2020 and as early as 2011. Gaps in immunization are not limited to low vaccine coverage rates but also include shortcomings in administering the vaccine, defects in the cold chain, vaccine refusal by parents, or immune amnesia [13,18]. The measles virus has only one serotype, eight different clades (A-H), and 24 genotypes for tracking and surveillance purposes. These genetic differences are superficial and do not change the virus’s fundamental structure. All currently available measles vaccines are derived from genotype A [22]. Despite this, studies consistently confirm that they induce a broad, cross-protective immune response that neutralizes all known circulating genotypes, including B3, D4, D8, and H1 [22,23,24]. A 2025 study published by the China CDC, for example, showed that cross-neutralization activity against different genotypes varied by less than 6.4-fold, a testament to the vaccine’s broad protective immunity [23]. Therefore, outbreaks of measles and measles breakthrough infections are most likely due to waning immunity, vaccine failure, and failure to vaccinate rather than emergence of a new clade.

Males consistently accounted for a slightly higher proportion of cases than females across all study years. This pattern may reflect the sex distribution of the Iraqi population (50.5% male). Additional factors may include sociocultural norms that result in greater social interaction among males and differences in hygiene-related practices compared with females.

Despite the recent surge in cases, there were 27 districts with low reporting distributed in Duhok, Erbil, Sulaymaniya, Anbar, Salahaldin, and Babylon. The consistent low reporting from Duhok, Erbil, and Sulaymaniya could be due too few human resources and work days which underestimate the magnitude of the rise in measles cases in those areas.

Although vaccination protects against all clades, the recent surge in measles cases in Iraq and globally indicates the need for molecular characterization of the measles virus. In addition, given recent findings of possible differences in the neutralizing potential of antibodies produced by one clade against other clades, the immunization coverage standard requires 96–98% to interrupt the transmission [3,25,26,27,28].

This study has several strengths. First, it is the first study to describe the epidemiology of the recent surge in measles cases in Iraq and provide insight into the demographic characteristics surrounding the increase. Second, this is the first study to utilize cluster analysis to determine high and low reporting, by districts, which may reflect the relationship with immunization coverage. Findings from cluster analysis help direct future immunization and surveillance awareness activities to cover both high and low clustering. In addition, these findings do not mean that other non-significant areas should be excluded from such activities but, rather, focusing on specific activities, such as active case detection, clinicians’ awareness campaigns, and surveillance evaluation should be encouraged in low clusters to detect and estimate underreporting. In contrast, focusing on immunization enhancement and case management in higher cluster areas might be top priorities. In addition, identifying clusters of high and low reporting can assist both the immunization program and the surveillance unit in identifying high-risk areas and planning targeted public health interventions. Specifically, areas with high vaccination coverage but a high number of measles cases may indicate problems related to the cold chain or vaccine administration practices. Conversely, areas with low vaccination coverage and high measles reporting clearly require strengthened immunization services. Areas characterized by both low vaccination coverage and low measles reporting may indicate gaps in surveillance, a need for additional training in vaccination and case detection, or the necessity for community awareness campaigns to improve vaccine acceptance.

Several limitations should be considered when interpreting the findings of this study. These include the lack of data on clinical presentation, possible complications, and case management. The availability of such information would have allowed for a better understanding of changes in measles clinical presentation, the burden of complications, and the potential impact of clinical management on reducing morbidity and mortality. Additional limitations include possible underreporting or surveillance bias, changes in healthcare-seeking behavior following the COVID-19 pandemic, variability in diagnostic confirmation practices between years, and the ecological nature and inherent constraints of spatial cluster analysis. Finally, delays in integrating district-level immunization coverage data with measles case counts at the time of analysis limited the ability to fully explain the observed patterns of high and low reporting clusters.

We recommend strengthening the surveillance system by improving clinicians’ awareness and understanding of the surveillance case definitions and emphasizing the importance of timely and complete reporting. In addition, we recommend integrating multiple data sources, including immunization records, case investigation forms, surveillance forms, and laboratory reports, to improve data completeness and accuracy.

Systematic documentation of the public health response to measles case surges is recommended to support the training of frontline healthcare workers and residents involved in community medical boards. Enhancing overall knowledge and capacity in infectious disease recognition, prevention, and response at both the healthcare and community levels will contribute significantly toward measles elimination efforts.

Further, we recommend expanding laboratory testing to two more public health laboratories to act as a reference for nearby governorates instead of having all samples tested at the CPHL during future outbreaks. These two additional laboratories should still submit the results to the CPHL and the Surveillance Section. In addition, the CPHL should verify the accuracy of a sample of the results for quality control. The use of advanced modelling methods to predict when and where the next outbreak of measles will be as part of routine surveillance work will help in modifying immunization plans to prevent or mitigate a possible outbreak as part of preparedness efforts. Lastly, documenting measles complications and both morbidity and mortality is essential in understanding the burden accurately.

## Figures and Tables

**Table 1:** Surveillance case definition of measles as per the measles and rubella field guideline in 2023 in Iraq.

Category	Definition/Criteria
Suspected Case Definition (Case Finding)	Any patient with fever and generalized maculopapular (non-vesicular) rash OR any person in whom a physician suspects measles
Laboratory-Confirmed Measles	A suspected case confirmed positive for measles IgM antibodies by testing at the Central Public Health Laboratory (CPHL), with any vaccine-associated illness ruled out
Epidemiologically Linked Measles	A suspected case not laboratory-confirmed but geographically and temporally related to a laboratory-confirmed or epidemiologically linked measles case, with rash onset dates occurring 7–23 days apart
Clinically Compatible Measles	A suspected case with fever and maculopapular (non-vesicular) rash and at least one of the following: cough, coryza, or conjunctivitis, with no adequate specimen collected and no epidemiologic link to a laboratory-confirmed measles case or other communicable disease
Non-Measles Non-Rubella Discarded Case	A suspected case that has been investigated and discarded as measles and rubella when any of the following apply: Negative CPHL laboratory test from an adequate specimen collected during the appropriate period after rash onset Epidemiologic linkage to a laboratory-confirmed outbreak of another communicable disease (not measles) Confirmation of another etiology Failure to meet the clinically compatible measles case definition If the case is also negative for rubella, this is non-measle/non-rubella case and is not included

**Table 2:** Characteristics of reported measles cases in Iraq, 2018–2019 and 2023–2024.

	2018	2019	2023	2024
	N (%)	N (%)	N (%)	N (%)
	1044	4586	14,301	33,048
**Diagnostics**				
Clinical	93 (8.9%)	3400 (74.1%)	9669 (78.4%)	31,361 (94.9%)
Laboratory confirmed	473 (45.3%)	1186 (25.9%)	2662 (21.6%)	1673 (5.1%)
**Sex**				
Male	288 (50.8%)	2413 (52.6%)	6462 (52.4%)	17,191 (52.0%)
Female	278 (49.2%)	2173 (47.4%)	5869 (47.6%)	15,857 (48.0%)
Age groups *				
Younger than 1 year	110 (19.4%)	1080 (23.5%)	2381 (19.3%)	7831 (23.7%)
1–4	293 (51.8%)	1425 (31.1%)	4954 (40.2%)	10,709 (32.4%)
5–14	90 (15.9%)	1016 (22.1%)	4045 (32.8%)	9905 (30.0%)
15+	67 (11.8%)	1065 (23.2%)	951 (7.7%)	4603 (13.9%)
**Vaccination status (Yes)**	NA	190 (4.1%)	552 (4.5%)	1545 (4.7%)
History of contact	NA	NA	163 (1.3%)	387 (1.2%)
Deaths	0	0	8 (0.06%)	52 (0.2%)

* The chi-square statistic is 1111.2463. The *p*-value is <0.00001. The result is significant at *p* < 0.05.

**Table 3:** Population-adjusted measles incidence rates in Iraq 2018–2019 and 2023–2024.

Year	Measles Incidence per 100,000 Population
2018	2.6
2019	11.1
2023	31.7
2024 *	71.8

* Incidence for 2024 reflects cases reported through May 19 and likely underestimates the full-year rate.

## Data Availability

The data that support the findings of this study are available from the corresponding author upon reasonable request.

## References

[1] Franconeri L., Antona D., Cauchemez S., Lévy-Bruhl D., Paireau J. (2023). Two-dose measles vaccine effectiveness remains high over time: A French observational study, 2017–2019. Vaccine.

[2] Siddiqui E., Khan M.S., Chandani D.K., Khalid M., Waafira A. (2025). Measles resurgence in Texas: A public health wake-up call. Ann. Med. Surg..

[3] Coughlin M.M., Beck A.S., Bankamp B., Rota P.A. (2017). Perspective on Global Measles Epidemiology and Control and the Role of Novel Vaccination Strategies. Viruses.

[4] Branda F., Giovanetti M., Petrosillo N., Ahmed M.M., Perra M., Sanna D., Ceccarelli G., Ciccozzi M., Bucci E., Scarpa F. (2025). Measles and public health: An integrative approach. Biol. Direct.

[5] Kulkarni R.D., Ajantha G.S., Kiran A.R., Pravinchandra K.R. (2017). Global eradication of measles: Are we poised?. Indian J. Med. Microbiol..

[6] World Health Organization (WHO) Measles Reported Cases and Incidence. https://immunizationdata.who.int/global/wiise-detail-page/measles-reported-cases-and-incidence?CODE=Global&YEAR=.

[7] Iraqi Ministry of Health (MoH Iraq) (2020). Annual Statistical Report 2020.

[8] World Health Organization (2025). Measles Deaths down 88% Since 2000, but Cases Surge. https://www.who.int/brunei/news/detail-global/28-11-2025-measles-deaths-down-88--since-2000--but-cases-surge.

[9] World Health Organization (2025). WHO: Measles Deaths Dropped by 88% in Past 25 Years, but Cases Now Surging.

[10] World Health Organization (2025). WHO Report Finds Measles Cases Are Surging Despite Drop in Deaths.

[11] World Health Organization (2025). Measles Cases in 2024 Increased by 86% in the WHO Eastern Mediterranean Region.

[12] Centers for Disease Control and Prevention (2024). Global Measles Outbreaks.

[13] Iraqi Ministry of Health (MoH Iraq) (2021). Annual Statistical Report 2021.

[14] Rabaan A.A., Al Mutair A., Alhumaid S., Garout M., Alsubki R.A., Alshahrani F.S., Alfouzan W.A., Alestad J.H., Alsaleh A.E., Al-Mozaini M.A. (2022). Updates on Measles Incidence and Eradication: Emphasis on the Immunological Aspects of Measles Infection. Medicina.

[15] Sbarra A.N., Mosser J.F., Jit M., Ferrari M., Ramshaw R.E., O’COnnor P., Krause L.K., Rogowski E.L.B., Portnoy A. (2023). Estimating national-level measles case-fatality ratios in low-income and middle-income countries: An updated systematic review and modelling study. Lancet Glob. Health.

[16] Ristić M., Milošević V., Medić S., Malbaša J.D., Rajčević S., Boban J., Petrović V. (2019). Sero-epidemiological study in prediction of the risk groups for measles outbreaks in Vojvodina, Serbia. PLoS ONE.

[17] Parums D.V. (2024). A Review of the Resurgence of Measles, a Vaccine-Preventable Disease, as Current Concerns Contrast with Past Hopes for Measles Elimination. Med. Sci. Monit..

[18] Fragkou P.C., Thomas K., Sympardi S., Liatsos G.D., Pirounaki M., Sambatakou H., Marantos T., Karofylakis E., Dourakis S.P., Tsiodras S. (2020). Clinical characteristics and outcomes of measles outbreak in adults: A multicenter retrospective observational study of 93 hospitalized adults in Greece. J. Clin. Virol..

[19] WHO/UNICEF (2025). European Region Reports Highest Number of Measles Cases in More Than 25 Years.

[20] Ghana News Agency (2025). Number of Measles Cases in Europe, Central Asia Highest in 27 Years.

[21] European Centre for Disease Prevention and Control (2025). Measles—Annual Epidemiological Report for 2024.

[22] Pan American Health Organization (2025). Measles Cases Rise in the Americas in 2025.

[23] Becker’s Hospital Review (2025). Measles Cases Double 2024 Total: 3 Updates.

[24] Cui X., Li Y., Yang Y., Tang W., Li Z., Chen H., Li Y., Cui X., Huang Z., Sun X. (2022). Charact eristics and Genomic Diversity of Measles Virus from Measles Cases with Known Vaccination Status in Shanghai, China. Front. Med..

[25] (2025). Measles Cases Spiking Across Southwest U.S. States. https://yalehealth.yale.edu/sites/default/files/2026-02/ysph%20vmoc%20special%20report%20-%20measles%20outbreak%20%20southwest%20us%205-21-2025.pdf.

[26] The Week (2025). India Among Top Countries Hit by Rising Measles Cases, WHO Report Finds.

[27] Kazinform (2024). Over 1,000 Measles Cases Recorded in Astana Since Beginning of Year.

[28] WHO/UNICEF (2026). Measles Cases Dropped in Europe and Central Asia in 2025 Compared to the Previous Year, but the Risk of Outbreaks Remains.

